# Melanocortin receptor 3 and 4 mRNA expression in the adult female Syrian hamster brain

**DOI:** 10.3389/fnmol.2023.1038341

**Published:** 2023-02-23

**Authors:** Megan A. L. Hall, Abigail L. Kohut-Jackson, Anna C. Peyla, Gloria D. Friedman, Nicole J. Simco, Johnathan M. Borland, Robert L. Meisel

**Affiliations:** Department of Neuroscience, University of Minnesota, Minneapolis, MN, United States

**Keywords:** melanocortin-3 receptor, melanocortin-4 receptor, *in-situ* hybridization (RNA-ISH), Syrian hamster, CNS distribution, energy homeostasis

## Abstract

Melanocortin 3 receptors (MC3R) and melanocortin 4 receptors (MC4R) are vital in regulating a variety of functions across many species. For example, the dysregulation of these receptors results in obesity and dysfunction in sexual behaviors. Only a handful of studies have mapped the expression of MC3R and MC4R mRNA across the central nervous system, with the primary focus on mice and rats. Because Syrian hamsters are valuable models for functions regulated by melanocortin receptors, our current study maps the distribution of MC3R and MC4R mRNA in the Syrian hamster telencephalon, diencephalon, and midbrain using RNAscope. We found that the expression of MC3R mRNA was lowest in the telencephalon and greatest in the diencephalon, whereas the expression of MC4R mRNA was greatest in the midbrain. A comparison of these findings to previous studies found that MC3R and MC4R expression is similar in some brain regions across species and divergent in others. In addition, our study identifies novel brain regions for the expression of MC3Rs and MC4Rs, and identifies cells that co-express bothMC3 and MC4 receptors within certain brain regions.

## 1. Introduction

Melanocortin receptors 3 and 4 (MC3/4R) were discovered by Gantz et al. (1993a,b) using human cDNA. The melanocortin system includes five g-protein coupled receptors, with only MC3R and MC4R expressed in the central nervous system, along with their endogenous agonist alpha melanocortin stimulating hormone (alpha-MSH), and antagonist (agouti-related protein) (Ellacott and Cone, 2006). Functionally, MC3Rs regulate body fat to mass ratio. For example, knockout of the MC3R gene in mice results in an increase in body fat mass with a corresponding decrease in lean mass. Melanocortin 3 receptor knockouts have also resulted in a decrease in anxiety-like behaviors (Butler et al., 2000; Chen et al., 2000; Ghamari-Langroudi et al., 2018; Sweeney et al., 2021). These effects of energy regulation in humans have recently been discovered, as mutations that cause a loss of function in MC3R result in a delayed onset of puberty and reduced height (Lam et al., 2021). MC3R is also involved in cardiovascular regulation, thermoregulation, and neuroendocrine processes (Roselli-Rehfuss et al., 1993). Melanocortin receptor 4 regulates food intake and energy expenditure (Balthasar et al., 2005; Tao, 2010). For example, administration of MC4R antagonists or the knockout of the MC4R gene in rodents resulted in an increase in feeding behavior, obesity, and linear growth, the opposite of what is seen with loss of MC3R function (Fan et al., 1997; Huszar et al., 1997; Tao, 2010). Melanocortin receptor 4 also has a role in sexual behaviors through the modulation of luteinizing hormone and prolactin surges in female rodents and erectile activity in male rodents (Van der Ploeg et al., 2002; Tao, 2010).

The distribution of MC3R and MC4R expression in the brain has been mapped by Roselli-Rehfuss et al. (1993), Mountjoy et al. (1994), and Kishi et al. (2003) in rats, and Gantz et al. (1993a,b), Liu et al. (2003), and Sweeney et al. (2021) in mice. Furthermore, a study was recently published that provided a more extensive description of the distribution of the cells that express MC3R mRNA in the central nervous system of mice (Bedenbaugh et al., 2022). The expression of MC3R tends to be isolated in the hypothalamus and thalamus in mice and rats (Roselli-Rehfuss et al., 1993; Bedenbaugh et al., 2022). On the other hand, MC4Rs are expressed more broadly across the central nervous system in mice and rats (Mountjoy et al., 1994; Liu et al., 2003; Tao, 2010). Because determining the distribution of the expression of MC3R and MC4R mRNA in the brain provides a key to understanding the function of this system, additional studies are needed to determine the degree of convergence and differences in the receptor distribution among species. Finally, no studies have simultaneously mapped and compared both MC3R and MC4R mRNA. Thus, this study analyzed the mapping of MC3R and MC4R mRNA across the central nervous system of adult Syrian hamsters, broadening the species studied and advancing our understanding of the relationship between MC3R and MC4R expression.

The utilization of RNAscope and imaging software can be advantageous by detecting expression at lower levels than prior methods, assessing expression and changes in expression across more fine-tuned subregions, and assessing the co-localization/co-expression of MC3R and MC4R mRNA. As an initial approach to cataloging the expression of MC3R and MC4R in the hamster brain, we chose to focus on the telencephalon, diencephalon, and midbrain. We observed expression in 26 brain regions across these levels of the brain. We chose to categorize the distribution within these three levels of the nervous system due to their functionality and evolutionary conservation. Our findings bring novel insights of the distribution and the expression of MC3R and MC4R mRNA in the brain.

## 2. Methods

### 2.1. Subjects

Adult female (*n* = 4) Syrian hamsters (*Mesocricetus auratus*) were purchased from Charles River Laboratories (Wilmington, MA, United States) at approximately 60 days of age (120–130 g). Females were housed individually in polycarbonate cages (50.8 × 40.6 × 20.3 cm). All animals were maintained on a reversed 14 h:10 h light/dark photoperiod (lights off at 1300 h). The animal room was maintained at a controlled temperature of 22°C, and food and water were available *ad libitum*. Subjects were ovariectomized (at approximately 90 days of age) and were approximately 140–150 days old at the time of tissue collection. All animal procedures were carried out in accordance with the National Institutes of Health Guide for the Care and Use of Laboratory Animals (NIH Publications No. 80–23; revised 2011) and approved by the University of Minnesota Institutional Animal Care and Use Committee.

### 2.2. Histology

Subjects were injected with a lethal dose of sodium pentobarbital (Beuthanasia-D, 0.25 ml i.p., Schering, Union, NJ, United States) and sacrificed by rapid decapitation. Brains were extracted, frozen in optimum cutting temperature compound (VWR Scientific Products) and stored at −80°C. Brains were later sliced coronally on a cryostat at 14 μm thickness and sections were mounted on Colorfrost Plus slides (Fisher Scientific, Waltham, MA, United States). Some studies in rats and mice have provided an extensive analysis of range of the distribution of melanocortin receptor expressing neurons. Here, we limited our focus to the forebrain and midbrain with tissue collected from slices containing the prefrontal cortex through to slices containing the dorsal raphe nucleus. Slides were stored at −80°C until RNAscope procedure treatment.

### 2.3. RNAscope

For *in-situ* hybridization, the protocols for fresh-frozen sample preparation and Multiplex fluorescent v2 assay were followed according to manufacturer’s instructions (Advanced Cell Diagnostics, Newark, CA, United States). Briefly, slides were removed from −80°C storage and fixed in 10% neutral buffered formalin (Sigma Aldrich, Burlington, MA, United States) for 15 min at 4°C.

Slides were rinsed twice in phosphate-buffered saline (9.8 g/l PBS), which contains 80 g/l sodium chloride, 14 g/l sodium phosphate dibasic, 3 g/l sodium phosphate monobasic, and 2 g/l potassium chloride (Fisher Scientific), at pH 7.4 and then dehydrated for 5 min each in 50, 70, 100, and 100% ethanol at room temperature. After air-drying for 5 min, hydrophobic barriers were drawn around the tissue to minimize loss of applied solutions. Slides were treated with Protease IV, incubated at room temperature for 30 min, and washed twice for 2 min in PBS. Two probes targeting Syrian hamster sequences were created by Advanced Cell Diagnostics (Newark, CA, United States) and used for the procedure: Mau-MC4R-C1 (mRNA encoding melanocortin-4 receptor, MC4R); GenBank accession number (XM_005074647.3, target nt region 519–1,483) and Mau-MC3R-C2 (mRNA encoding melanocortin-3 receptor, MC3R); GenBank accession number (XM_005074460.4, target nt region 932–1997). Probes were applied such that each tissue received 75 μl of solution that contained a 50:50 ratio of probe diluent (Advanced Cell Diagnostics) and probe mixture. Each probe mixture contained a 50:1 ratio of C1:C2 (each tissue received 37.50 μl of probe diluent, 36.75 μl of C1 and 0.75 μl of C2). Tissues were incubated at 40°C for 2 h. Following incubation, tissues were washed twice for at room temperature for 2 min each with washing buffer solution (8.7 g/l of sodium chloride, 4.41 g/l of sodium citrate, 3 g/l of sodium dodecylsulfate) based on Wang et al. (2012). Tissues were then incubated with amplifying probes (AMP1, AMP2, and AMP3, Advanced Cell Diagnostics) at 40°C for 30 min, 30 min, and 15 min, respectively. Slides were washed with washing buffer solution twice at room temperature for 2 min each between each incubation step. Fluorescently labeled probes (Akoya Biosciences, Marlborough, MA, United States) were applied with the following assignments: MC4R-C1 with Opal 570 nm, concentration 1:750, and MC3R-C2 with Opal 520 nm, concentration 1:750. TSA buffer (Advanced Cell Diagnostics) was mixed with each dye to achieve the desired dilution. For each fluorescent probe, slides were first incubated with horseradish peroxidase (HRP) (Advanced Cell Diagnostics) for the corresponding channel at 40°C for 15 min, then with the probe at 40°C for 30 min, and finally with HRP blocker (Advanced Cell Diagnostics) at 40°C for 15 min. Slides were washed twice with washing buffer solution for 2 min each between each incubation step. Slides were coverslipped with ProLong Gold Antifade Mountant (Advanced Cell Diagnostics) and stored at 4°C. An example of the staining can be seen in Supplemental Figure 1.

### 2.4. Confocal imaging

Experimenters scanned all the tissue slices with a fluorescent microscope to identify brain regions that expressed either MC3R or MC4R mRNA. Twenty-six brain regions were chosen based on findings in previous literature, functional sites of melanocortin action, and visual inspection of expression within tissue by the experimenter. The 26 brain regions were identified based on *A Stereotaxic Atlas of the Golden Hamster Brain* (Morin and Wood, 2001). Schematic diagrams from the hamster atlas were used at the level that corresponds to where the region of interest was imaged and overlayed with a color fill-in using BioRender to help illustrate the mRNA labeling within a region. The serial sections were taken at a specific location (matched across brains) within each brain region, as indicated by the atlas plates and represent a rostral-caudal distance of less than 0.5 mm. Anatomical abbreviations are as follows:

**Table:** 

ARC	Arcuate nucleus of the hypothalamus
BIC	Nucleus of the brachium of the inferior colliculus
BNST	Bed nucleus of the stria terminalis
CPu medial, lateral, dorsal	Caudate-putamen medial, lateral, dorsal
DEn	Dorsal endopiriform nucleus
DRN	Dorsal raphe nucleus
HPC	Hippocampus (dorsal/ventral CA1, CA2)
IL	Infralimbic cortex
LHb	Lateral habenula
LS	Lateral septum
MePD	Medial amygdaloid nucleus, Posterodorsal part
MD	Mediodorsal thalamic nucleus
MS	Medial septum
MPOA	Medial preoptic area
NAc core	Nucleus accumbens core
NAc shell	Nucleus accumbens shell
OT	Olfactory tubercle
PAG	Periaqueductal gray
PrL	Prelimbic cortex
PVN	Periventricular hypothalamus
VMH	Ventromedial hypothalamus
VTA	Ventral tegmental area

All images were acquired on a Leica TCS SPE confocal microscope under the same scanning parameters. An image was collected in the left and right hemisphere from two tissue sections (in series) for all brain regions for each subject (resulting in a sample of four images for each brain region for each subject). Images were collected with a 20X/0.60 advanced correction system objective with a pixel distribution of 1,024 × 1,024 at a frequency of 8 kHz. A solid-state laser with 488 and 532 nm wavelengths and an ultra-high dynamic PMT detector was utilized to capture z-stack images with 1.5 μm spacing for a maximum of 15 steps. The pinhole size was 1 airy unit (AU), 2 frames were averaged, and optical zoom was 1.00X. 3D images produced were 550 × 550 × 14 μm.

### 2.5. Image analysis

Images were analyzed using Imaris software (Oxford Instruments, version 9.7.2) to investigate (1) the number of puncta of mRNA, (2) the number of cells that express MC3R and MC4R mRNA, and (3) the colocalization of MC3R and MC4R mRNA. First, the Spots feature was utilized to create a model of each channel. The estimated XY diameter was set at 0.650 μm. Next, the “quality” filter was applied, and the threshold was adjusted manually to include all spots that met the diameter in each channel. Then the “average distance to 3 nearest neighbors” filter was applied for all spots that passed the quality filter. The number of spots that were filtered into the model was then recorded for each of the MC3R and MC4R channels (Supplemental Figures 2A–C).

The Surfaces feature was then utilized to create a secondary model of each channel to estimate the number of cells that express MC3R and MC4R mRNA. The threshold for absolute intensity was set with a seed point diameter of 15 μm. First, the “quality” filter was applied, and the threshold was adjusted manually to include a seed point for all possible cells in each channel. Second, the “area” filter was applied such that all cells with an area of 19.63 μm^3^ were included in the surface model. Third, a “volume” filter was applied in order to include in the model only cells with a volume greater than 65.45 μm^3^. The number of cells that were filtered into the model was then recorded for each of the MC3R and MC4R channels. The criteria for cell surfaces excluded any non-specific binding that may be present, as the non-specific binding would not cluster in a way that meets the area and volume parameters that we have set (Supplemental Figures 3A–F).

For co-expression analysis, a similar procedure was followed using Imaris software. Receptor expression from the image was filtered such that only the overlap between two channels of interest was displayed. The Surfaces feature was used to create a model of the new channel, again using “quality,” “area,” and “volume” filters. Only cells that met these criteria were counted as colocalized. The percent of total cells that co-expressed MC3Rand MC4R mRNA was calculated by the following formula: cells containing both MC3R and MC4R label/total numbers of cells containing MC3R and/or MC4R label X 100.

### 2.6. Plus scale conversion

The average number of cells expressing MC3R or MC4R mRNA (i.e., labeled cells) was calculated for each region and used to determine the strength of expression within a brain region (Table 1). The four images per region for all four subjects were averaged together. Five categories were created and are as follows: Regions that had an average of 0 cell count averages are categorized as lack of expression and labeled as −, 1–24 cell count averages are categorized as low and labeled as +, 25–49 cell count averages are categorized as moderate and labeled as + +, 50–74 cell count averages are categorized as high and labeled as + + +, and 75–100 cell count averages are categorized as highest and labeled as + + + +.

**Table 1:** Plus scale conversion.

Brain region	MC3R		MC4R	
	Avg. cell count number	Plus conversion	Avg. cell count number	Plus conversion
PrL	1	+	28.9	+ +
IL	0	−	47.2	+ +
NAc Core	0.8	−	15.3	+
NAc Shell	3.1	+	16.9	+
CPu medial	0	−	18.5	+
CPu lateral	0	−	5.9	+
CPu dorsal	0	−	11.9	+
OT	0	−	47.1	+ +
LS	45.6	+ +	20	+
MS	11.2	+	45.5	+ +
BNST	7.1	+	39.1	+ +
DEn	4.2	+	56.9	+ + +
MePD	6.3	+	47.8	+ +
Dorsal CA1	3.7	+	33.4	+ +
Dorsal CA2	2.1	+	27.3	+ +
MD	64.4	+ + +	0	−
LHb	46	+ +	10.3	+
MPOA	25.3	+ +	80.1	+ + + +
PVN	4.3	+	67.5	+ + +
VMH	83.9	+ + + +	8.7	+
ARC	37.8	+ +	22.7	+
Ventral CA1	1	+	90.1	+ + + +
PAG	19.3	+	67.9	+ + +
BIC	91.5	+ + + +	0	−
VTA	7.3	+	62.2	+ + +
DRN	3.8	+	67.1	+ + +

Average receptor cell count number conversion to qualitative plus scale. Averages were derived from the total cell counts across all the images divided by the number of images. Plus scale was determined using the lowest average and the highest average, with a range of 0 to 100 and greater. Five categories were created, one being – (lack of expression) and the other four ranging from + to ++++ (low to highest). The scale is as follows: 0 average cell counts are categorized as lack of expression and equal to –, 1–24 average cell counts are categorized as low and equal to +, 25–49 average cell counts are categorized as moderate and equal to + +, 50–74 average cell counts are categorized as high and equal to + + +, 75–100 average cell counts are categorized as highest and equal to + + + +.

## 3. Results

### 3.1. Telencephalon

**Figure 1:**
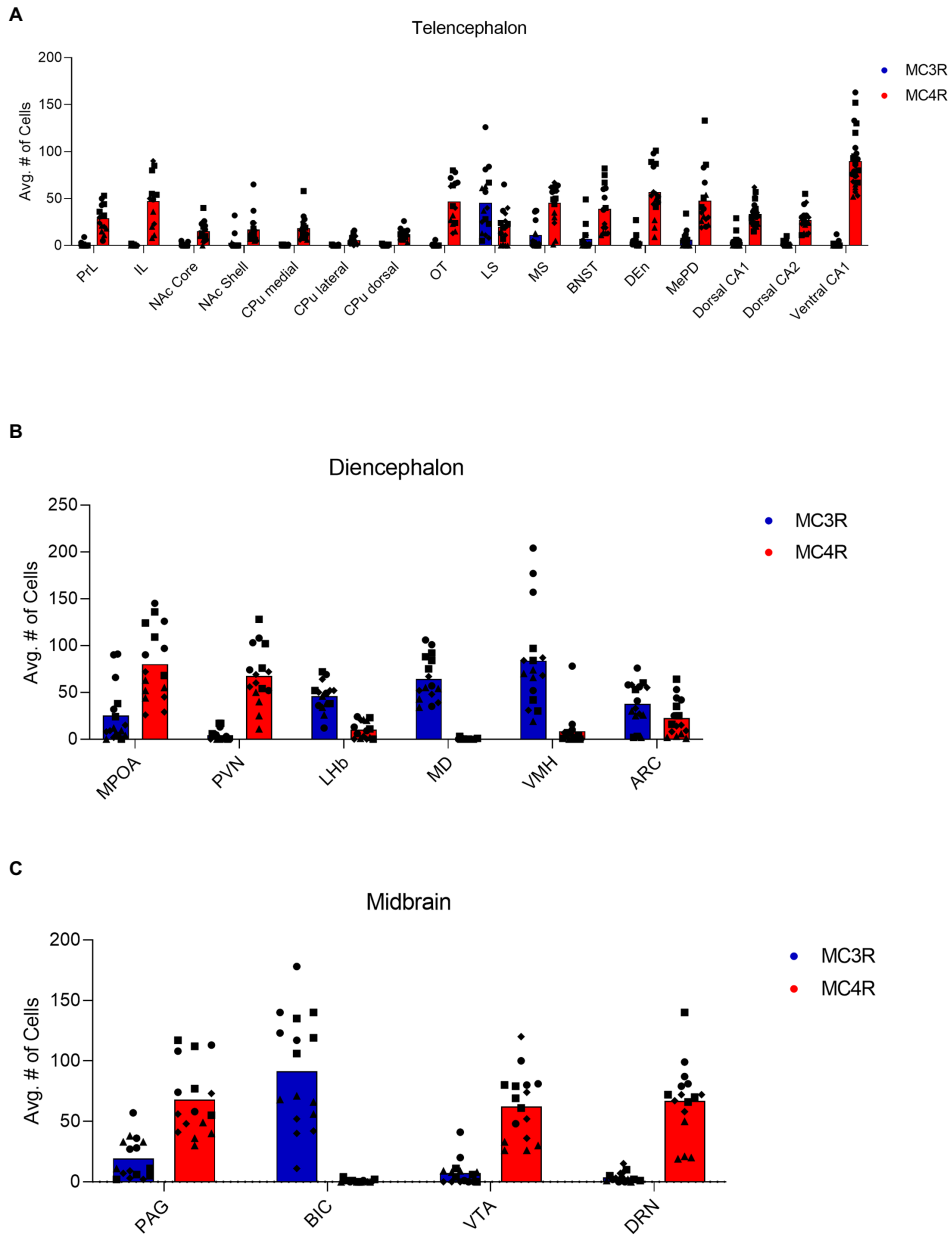
The number of cells that express MC3R mRNA (blue left-hand bar for each pair) and MC4R mRNA (red right-hand bar for each pair) in the regions of the **(A)** telencephalon, **(B)** diencephalon, and **(C)** midbrain. Each symbol indicates a section of the region from each subject. Circles indicate counts from subject one. Squares indicate counts from subject two. Triangles indicate counts from subject three. Diamonds indicate counts from subject four.

**Figure 2:**
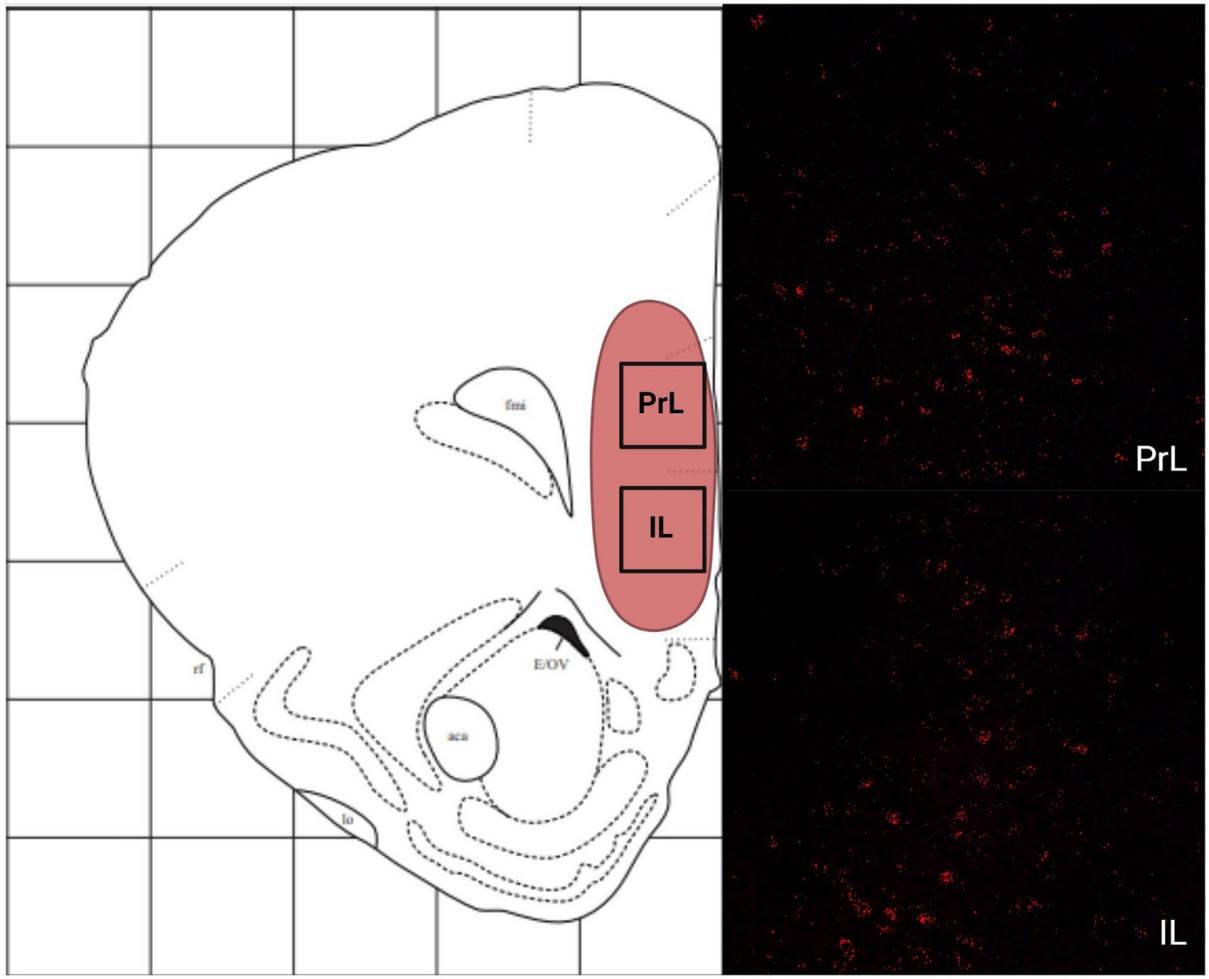
The expression of MC3R and MC4R mRNA in the IL and the PrL. For this and remaining figures: Coronal sections were used to estimate the expression of MC3R and MC4R mRNA (2–4 sections/hamster) using RNAscope. A schematic diagram outlining each region is shown on the left, followed by a representative RNAscope image on the right. Red overlay in the schematic depicts the general area of MC4R mRNA expression. Yellow overlay in the schematic depicts the general area of MC3R and MC4R mRNA expression. Blue overlay in the schematic depicts the general area of MC3R mRNA expression. Red labeling depicts MC4R mRNA. Blue labeling depicts MC3R mRNA. Created with BioRender.com

**Figure 3:**
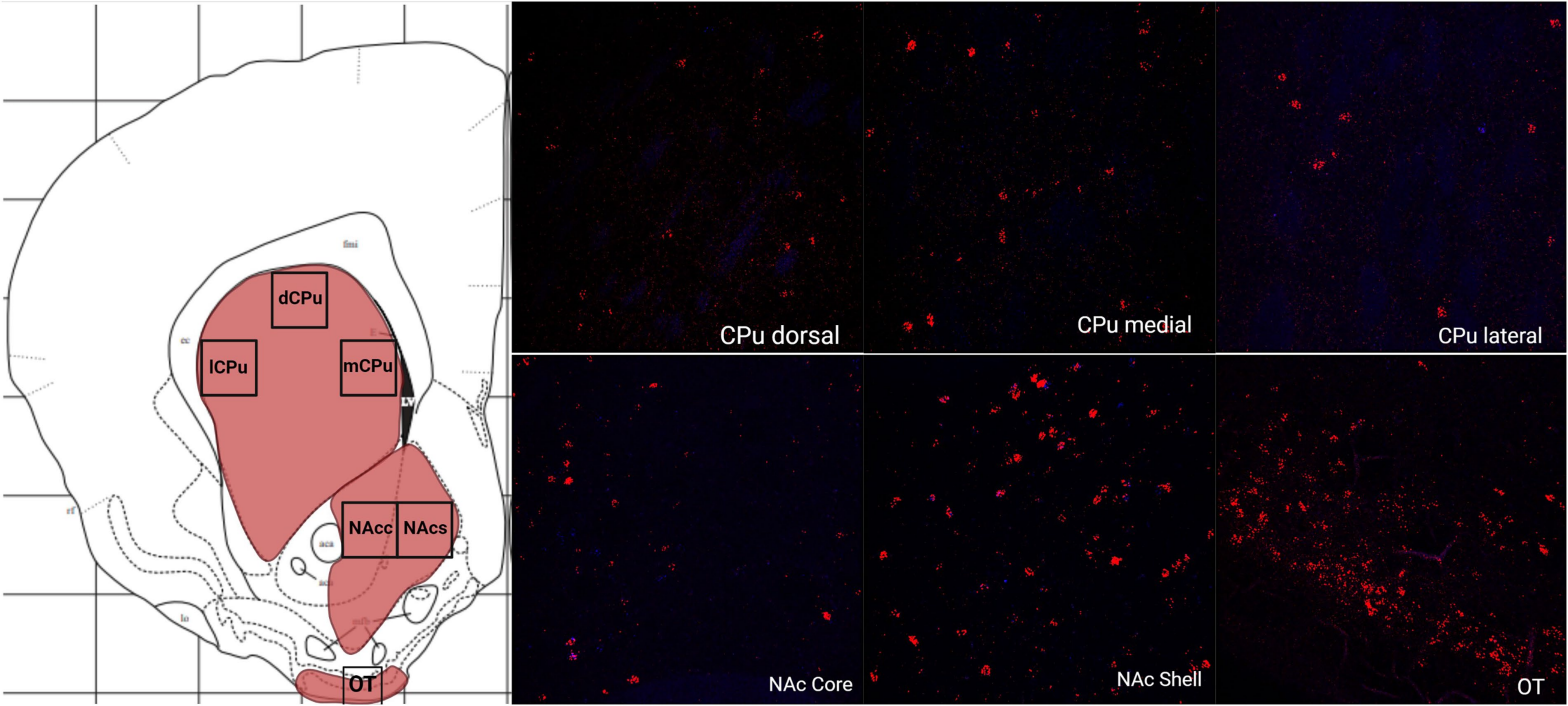
The expression of MC3R and MC4R mRNA in the dorsal, the lateral, and the medial CPu (dCPu, lCPu, and mCPu), the NAc core and shell (NAcc and NAcs), and the OT.

In the telencephalon, there was a greater number of cells that expressed MC4R mRNA than MC3R mRNA in all the regions analyzed except the LS (Figure 1A; Table 1). The number of cells that expressed MC3R mRNA within the PrL and NAc shell was low, with an average of 1 and 3.1 cell counts, respectively. The IL of the medial prefrontal cortex, the subregions of the CPu, the NAc core, and the OT all had a lack of cells that expressed MC3R mRNA (0 to 4 average cell counts; Figures 1A, 2, 3). In the PrL and the IL the number of cells that expressed MC4R mRNA was moderate, an average of 28.9 and 47.2 cell counts, respectively, per 550 × 550 μm sampling area (Figure 1A). In the NAc core and shell the number of cells that expressed MC4R mRNA was similar, but low; cell counts were 15.3 for the NAc core and 16.9 for the NAc shell (Figure 1A). Low numbers of MC4R cells were also observed in the medial, the lateral, and the dorsal CPu. The number of cells that express MC4R was three times greater in the medial CPu (23.9) compared to the lateral CPu (7.8) and slightly greater compared to the dorsal CPu (14.5; Figure 1A). The number of cells that express MC4R mRNA within the OT was moderate with an average of 47.1 cells (Figures 1A, 3).

**Figure 4:**
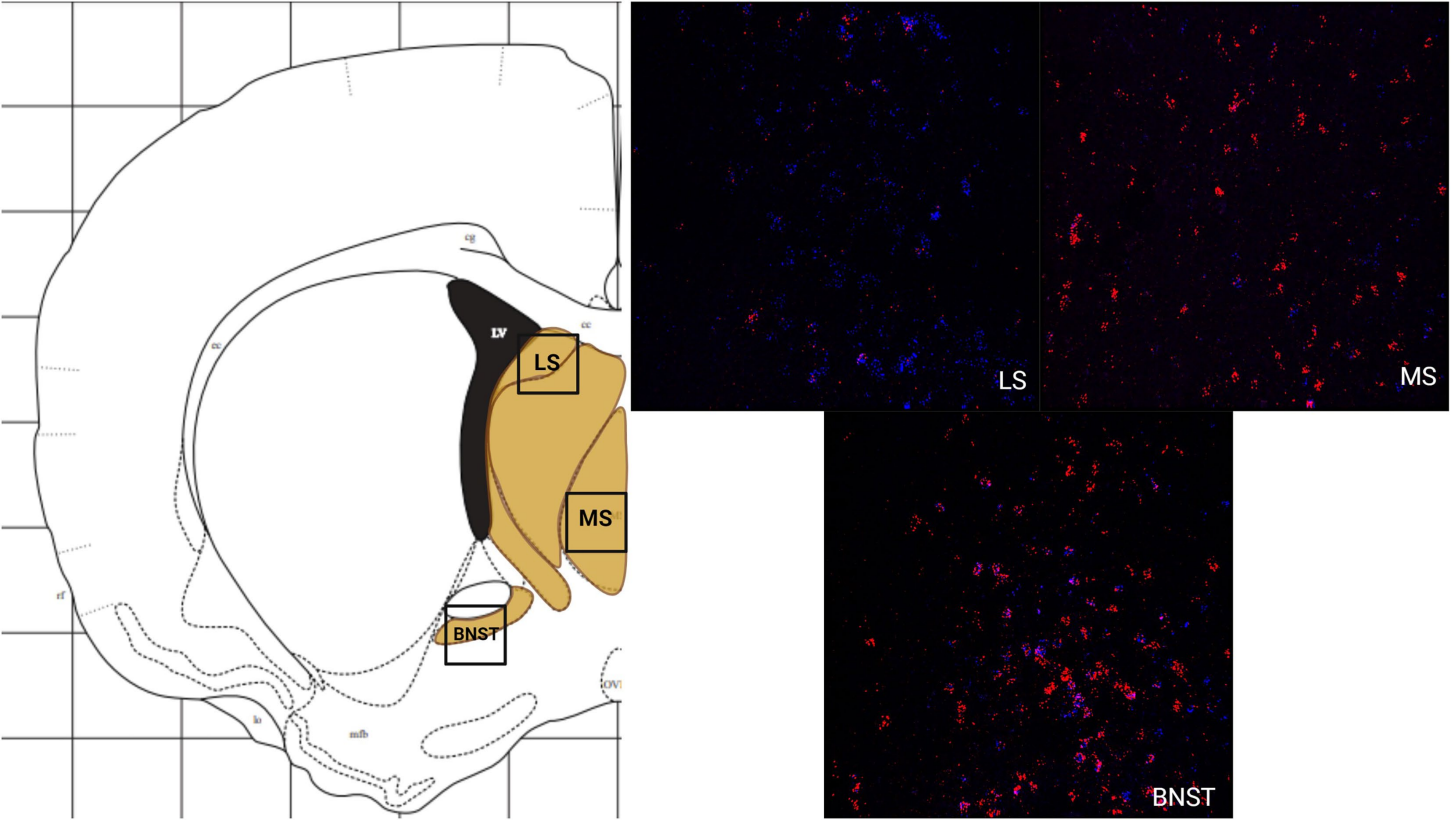
The expression of MC3R and MC4R mRNA in the LS, the MS, and the BNST.

The LS had a greater number of cells that express MC3R mRNA compared to MC4R mRNA, whereas the MS had a greater number of cells that express MC4R mRNA compared to the number of cells that express MC3R mRNA. The number of cells that express MC3R mRNA within the LS was moderate, with an average of 45.6 cell counts (Figure 1A). The number of cells that express MC4R mRNA within the LS was low (20 average cell counts; Figure 1A). The number of cells that express MC3R mRNA within the MS was low, with an average of 11.2 cell counts (Figure 1A). The number of cells that express MC4R mRNA within the MS was moderate, with an average of 45.5 cell counts (Figures 1A, 4). The number of cells expressing MC3R mRNA was four times greater in the LS compared to the MS, whereas the number of cells expressing MC4R mRNA was two times greater in the MS compared to the LS. The number of cells that expressed MC3R mRNA within the BNST was low (7.1 average cell counts), whereas the number of cells that expressed MC4R mRNA within the BNST was moderate (39.1 average cell counts; Figures 1A, 4).

**Figure 5:**
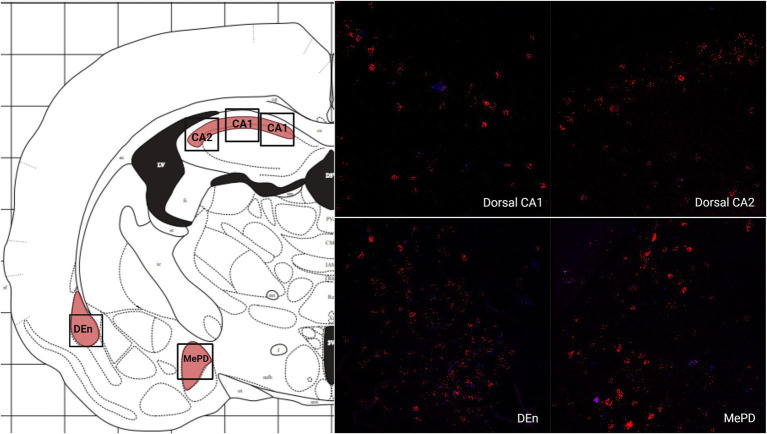
The expression of MC3R and MC4R mRNA in the Dorsal CA1, Dorsal CA2, DEn, and MePD.

**Figure 6:**
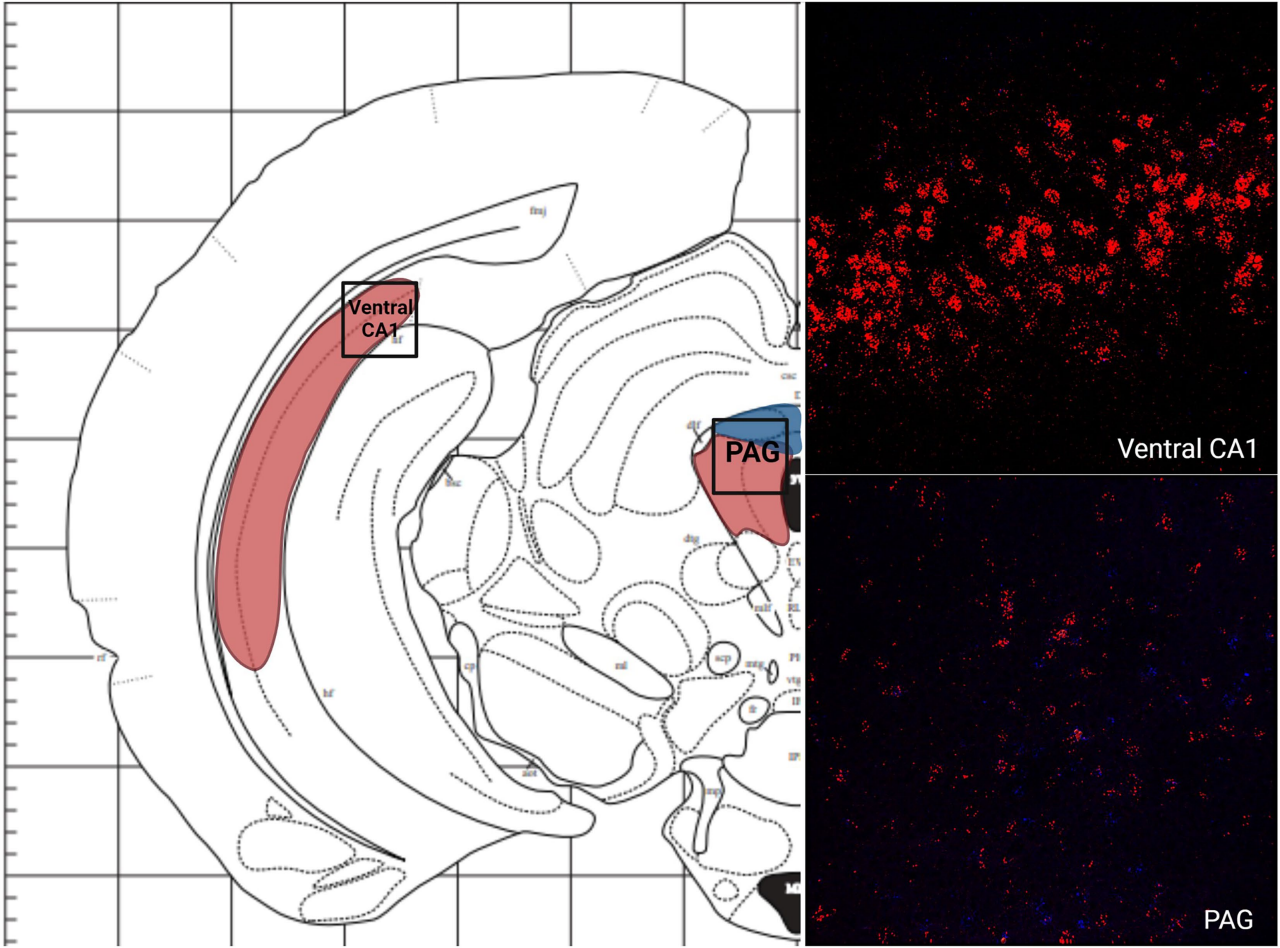
The expression of MC3R and MC4R mRNA in the MPOA and the PVN.

There was a low average number of cells that expressed MC3R within the DEn, while the number of cells that express MC4R mRNA was high (4.2 and 56.9 average cell counts respectively; Figures 1A, 5). The number of cells that express MC3R mRNA within the MePD of the amygdala was low (6.3 average cell counts), and the number of cells that express MC4R mRNA within the MePD of the amygdala was moderate (47.8 average cell counts; Figures 1A, 5). The number of cells that express MC3R mRNA in the dorsal CA1 and CA2 regions of the hippocampus was low; there was an average of 3.7 and 2.1 cell counts, respectively, (Figure 1A). The number of cells that express MC4R mRNA in the dorsal CA1 and CA2 regions of the hippocampus were similar and moderate, there was an average of 33.4 and 27.3 cell counts, respectively, (Figures 1A, 5). The ventral CA1 region of the hippocampus had a low average number of cells that expressed MC3R mRNA (an average of 1 cell; Figure 1A). The ventral CA1 region of the hippocampus had the highest number of cells that express MC4R mRNA compared to the other regions analyzed in the hamster (90.1 average cell counts; Figures 1A, 6).

### 3.2. Diencephalon

**Figure 7:**
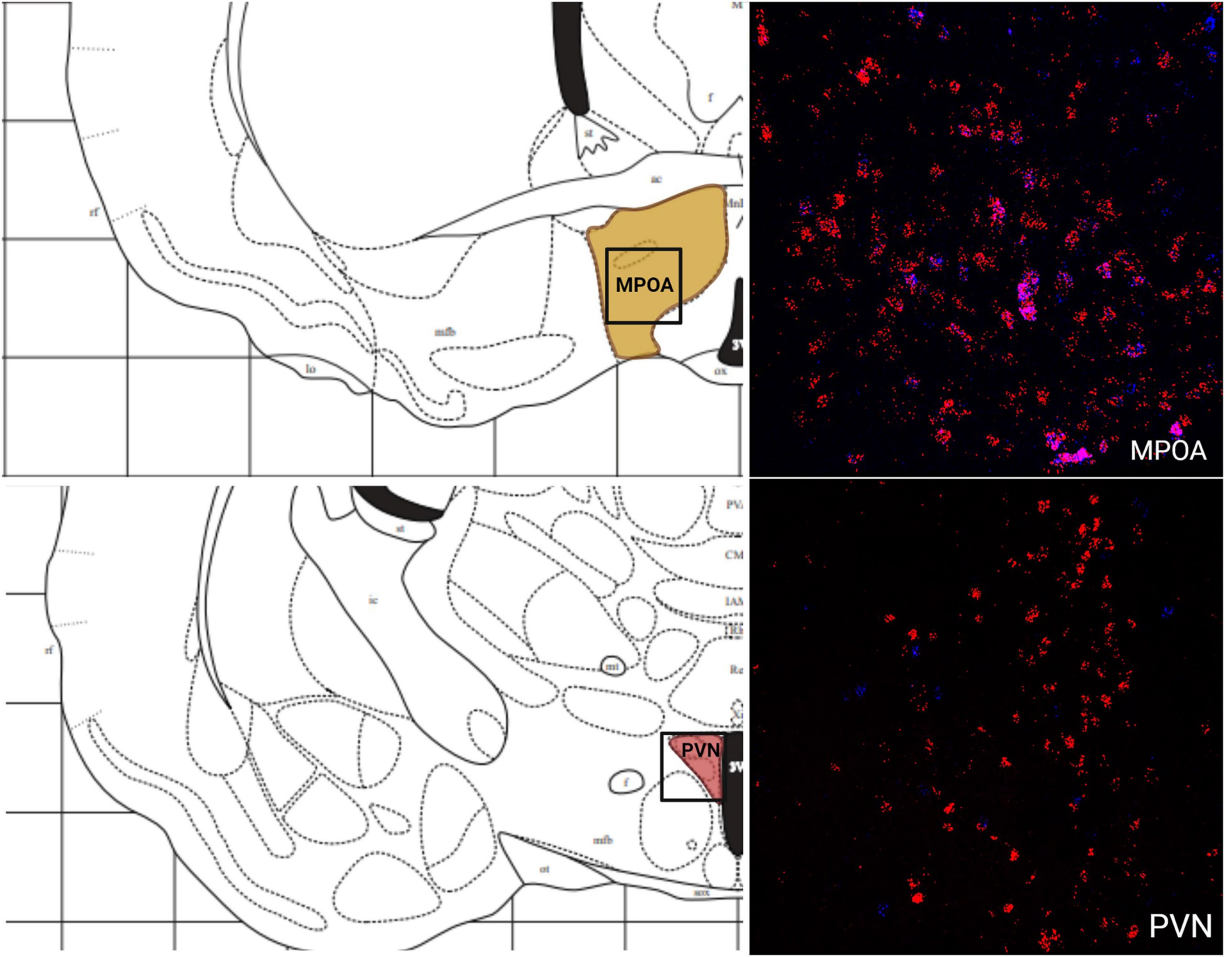
The expression of MC3R and MC4R mRNA in the LHb and the MD.

**Figure 8:**
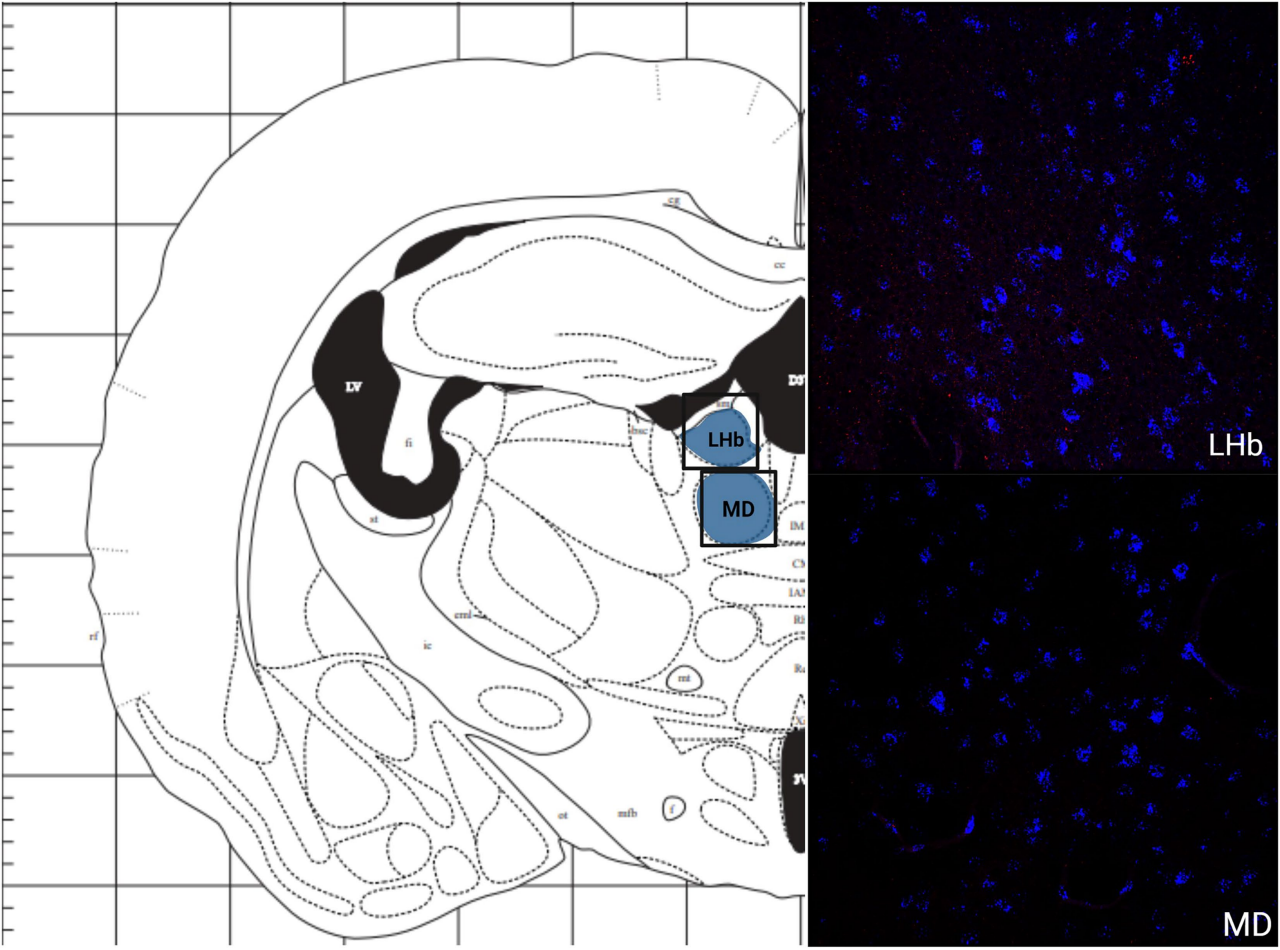
The expression of MC3R and MC4R mRNA in the VMH and the ARC.

**Figure 9:**
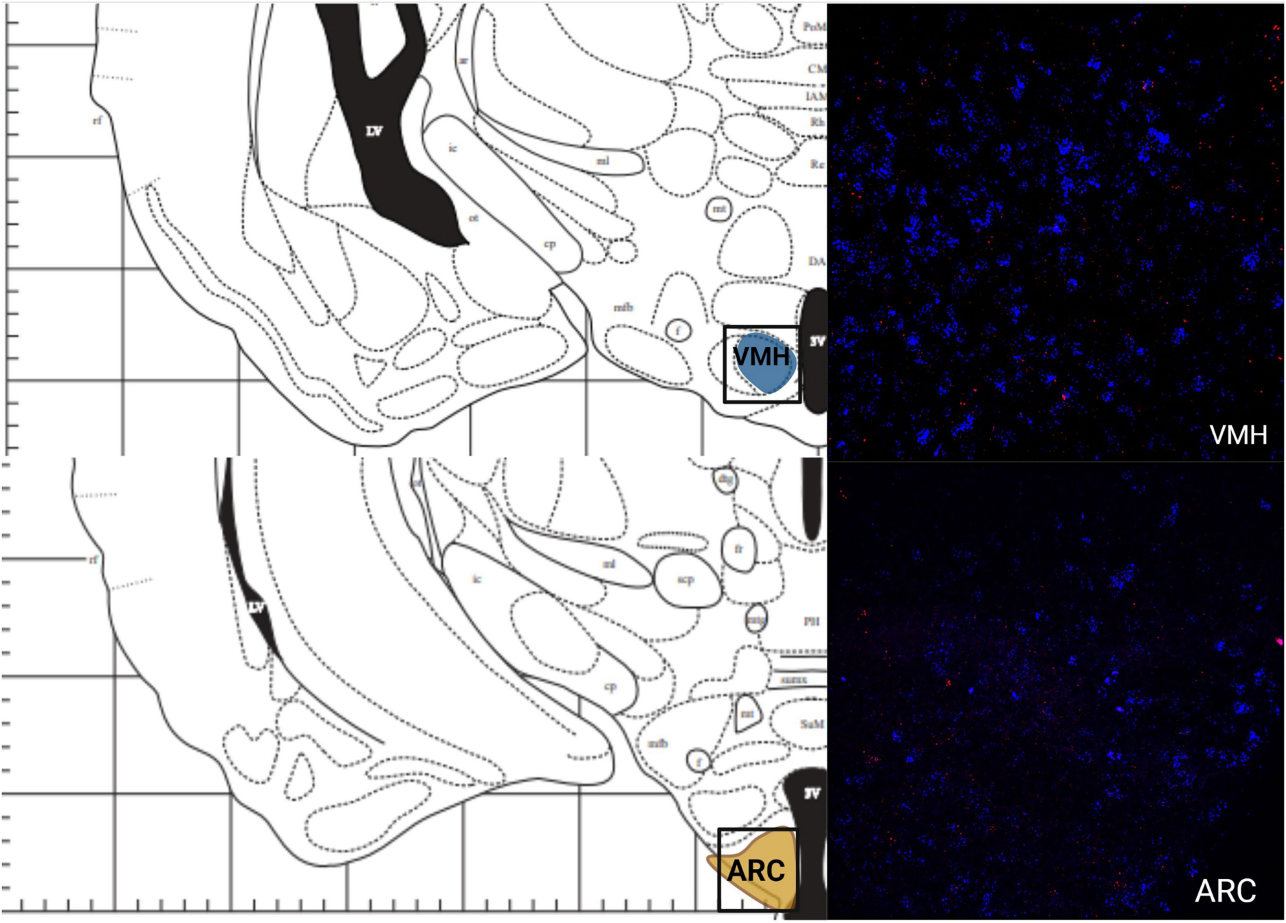
. The expression of MC3R and MC4R mRNA in the Ventral CA1 region of the hippocampus and the PAG.

The greatest MC3R hybridization was observed within subregions of the diencephalon (Figure 1B; Table 1). However, there was also substantial MC4R hybridization which varied according to diencephalic subregion. The number of cells expressing MC3R mRNA within the MPOA was moderate (25.3 average cell counts), whereas the number of cells expressing MC4R mRNA within the MPOA was highest (80.1 average cell counts; Figures 1B, 7). There was a low average number of cells expressing MC3R within the PVN (4.3 average cell counts), whereas the number of cells expressing MC4R mRNA in the PVN was high (67.5 average cell counts; Figures 1B, 7). Melanocortin receptor 4 hybridization was greatest within the parvocellular regions of the PVN. The number of cells expressing MC3R mRNA within the LHb was moderate (46 average cell counts), whereas the number of cells expressing MC4R mRNA in the LHb was low (10.3 average cell counts; Figures 1B, 8). The number of cells expressing MC3R mRNA within the MD was high, there was an average of 64.4 cell counts (Figure 8). There were no cells expressing MC4R mRNA within the MD (Figures 1B, 8). The VMH had the highest MC3R hybridization for the brain regions analyzed in the diencephalon. There was an average of 83.9 cells within the VMH that expressed MC3R mRNA (Figure 1B). The number of cells that expressed MC4R mRNA within the VMH was low (8.7 average cell counts; Figures 1B, 9). The number of cells expressing MC3R mRNA was moderate while the number of cells with MC4R mRNA expression was low in the ARC (37.8 and 22.7 average cell counts respectively; Figures 1B, 9).

### 3.3. Midbrain

**Figure 10:**
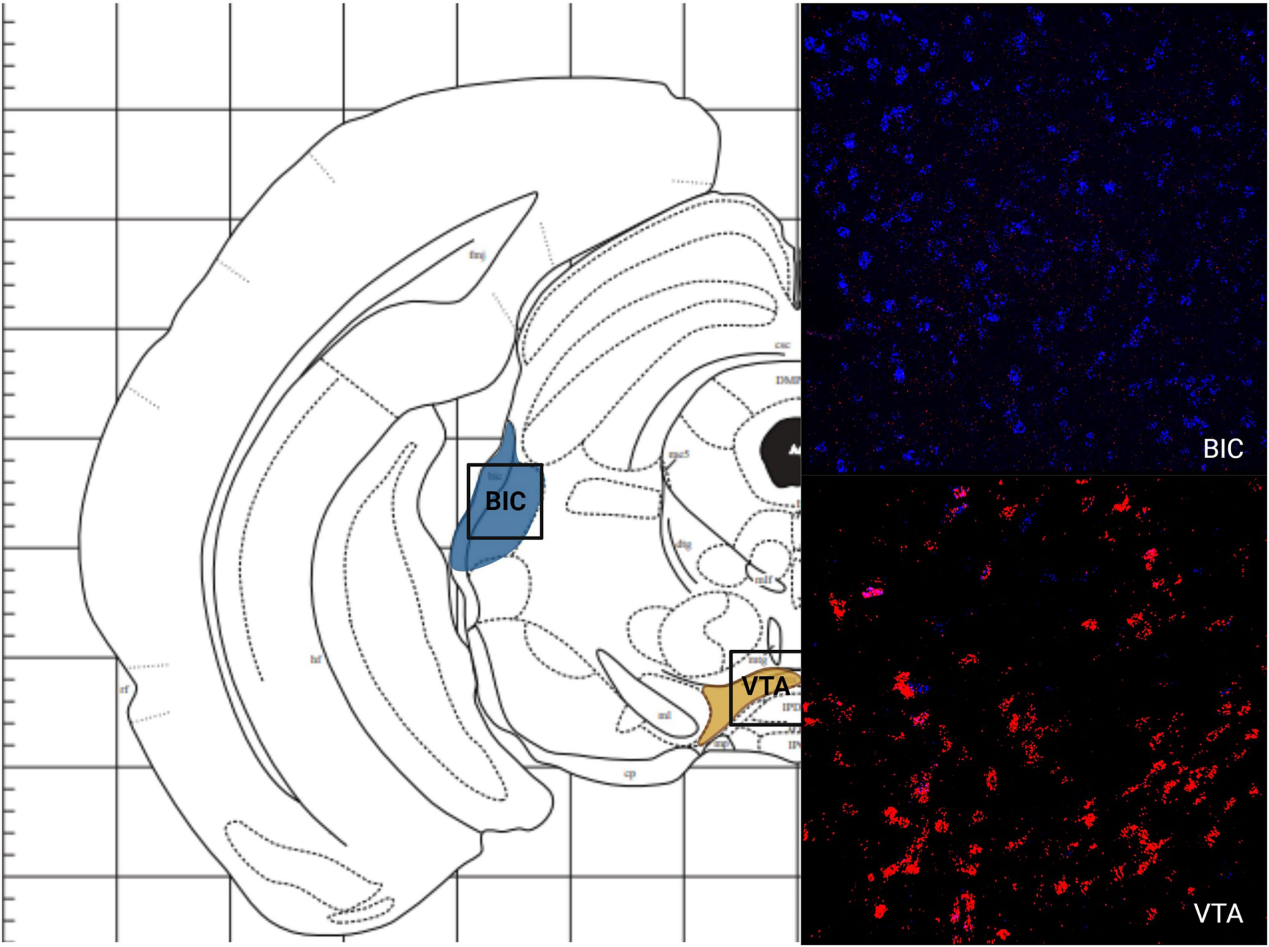
The expression of MC3R and MC4R mRNA in the BIC and the VTA.

**Figure 11:**
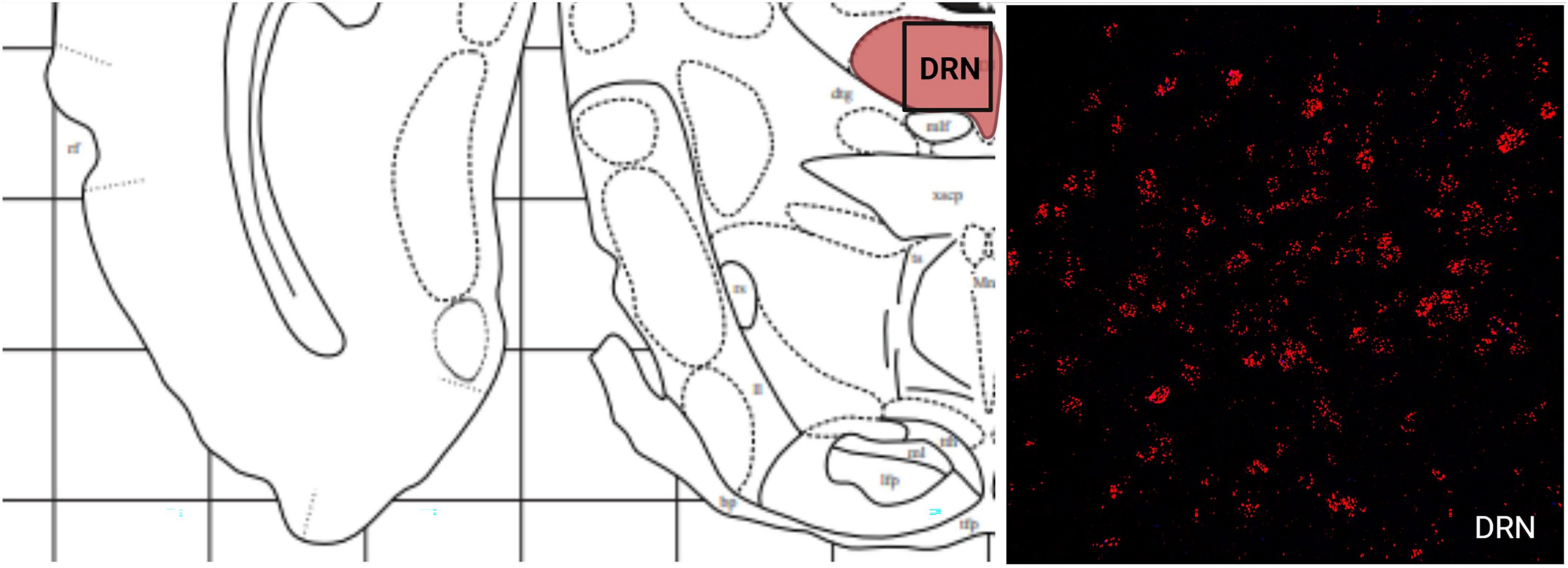
The expression of MC3R and MC4R mRNA in the DRN.

Like the telencephalon, the midbrain generally had higher expression of MC4R mRNA compared to the expression of MC3R mRNA (Table 1). The highest levels of MC3R hybridization across all the regions examined was within the midbrain (Figure 1C). The PAG exhibited low MC3R hybridization (19.3 average cell counts; Figure 6). The number of cells that express MC4R mRNA within the PAG was high, with an average of 67.9 cell counts (Figures 1C, 6). The BIC had the highest number of cells that express MC3R mRNA compared to the other regions analyzed in the hamster (91.5 average cell counts; Figure 1C). There was a lack of cells that express MC4R mRNA within the BIC (Figures 1C, 10). The number of cells that express MC3R mRNA within the VTA was low while the number of cells that express MC4R mRNA was high (7.3 and 62.2 average cell counts respectively; Figures 1C, 10). The DRN exhibited a low average of cells that expressed MC3R mRNA (3.8 average cell counts; Figure 1C). Finally, the number of cells that express MC4R mRNA within the DRN was high (67.1 average cell counts; Figures 1C, 11).

### 3.4. Colocalization

**Figure 12:**
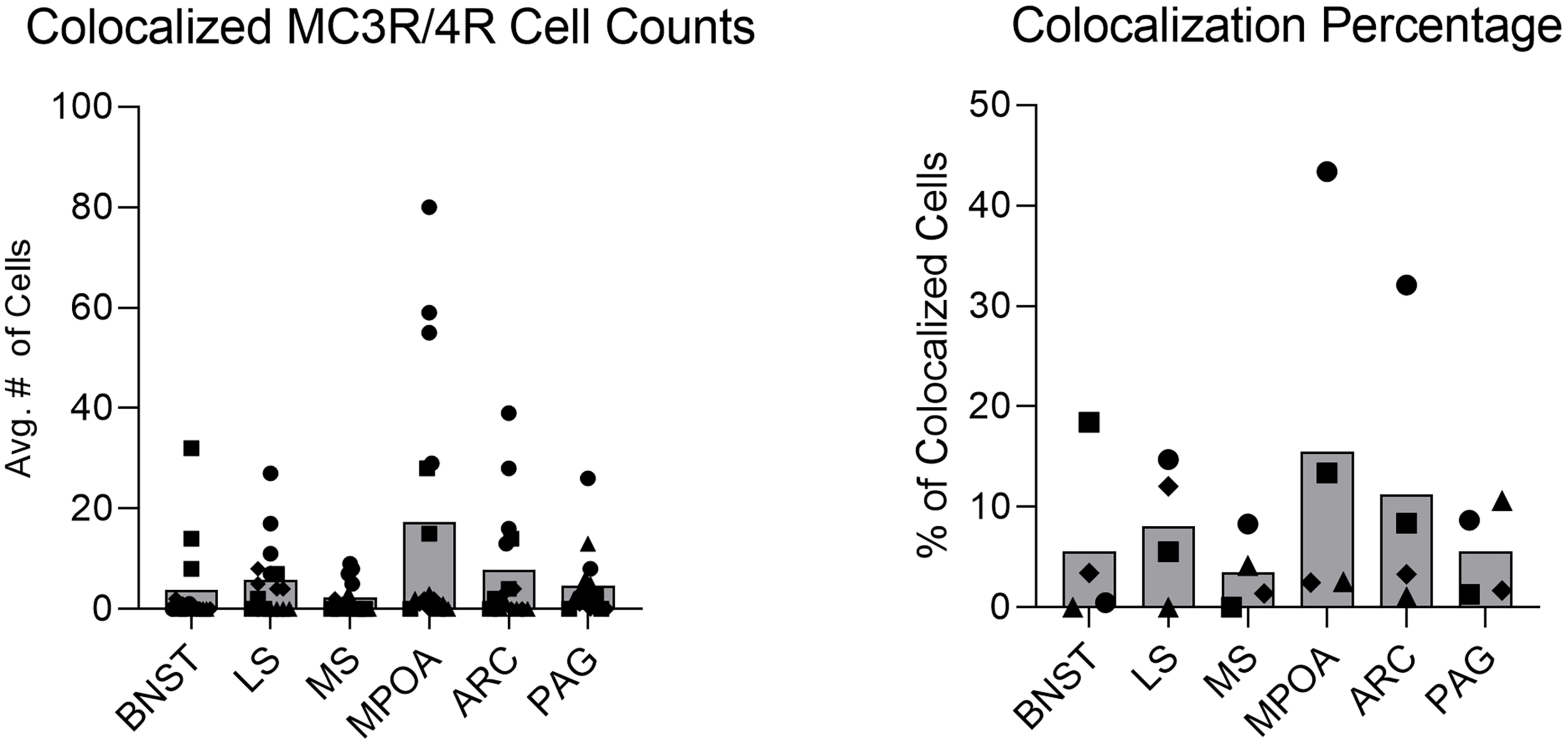
On the left: a colocalization graph illustrating the number of cells that co-express MC3R and MC4R mRNA in select brain regions. On the right: a colocalization percentage graph illustrating the percentage of cells that co-express MC3R and MC4R mRNA in select brain regions. Bars indicate the average number or percentage of cells that co-expressed MC3R and MC4R mRNA. Circles indicate counts from subject one. Squares indicate counts from subject two. Triangles indicate counts from subject three. Diamonds indicate counts from subject four.

The colocalization of MC3R and MC4R mRNA was conducted in the BNST, LS, MS, MPOA, ARC, and PAG. These regions demonstrated *in situ* hybridization signaling of both MC3R and MC4R. There was variability in overlap, though in general there was a relatively low level of colocalization. The percentage of colocalization for cells that expressed both MC3R and MC4R mRNA in the LS was 8.1%. The percentage of colocalization for cells that expressed both MC3R and MC4R mRNA in the MS was 3.5%. The percentage of colocalization for cells that expressed both MC3R and MC4R mRNA in the BNST was 5.6%, with the percentage of colocalization for cells that expressed both MC3R and MC4R mRNA in the PAG at 5.6% (Figure 12). The MPOA and the ARC exhibited the highest percentage of colocalization for cells that expressed both MC3R and MC4R mRNA compared to all the other brain regions analyzed. In the MPOA, 15.5% of the cells that expressed MC3R mRNA also co-expressed MC4R mRNA (Figure 12). In the ARC, 11.2% of the cells that expressed MC3R mRNA also co-expressed MC4R mRNA (Figure 12).

## 4. Discussion

In this study we mapped the expression of MC3R and MC4R mRNA across the central nervous system of Syrian hamsters. Overall, there is more widespread expression of MC4R mRNA in the hamster brain compared to the expression of MC3R mRNA. The expression of MC3R mRNA was contained primarily in the diencephalon. The expression of MC4R mRNA was greatest in the midbrain. These observations are consistent with those described in previous mapping studies (Roselli-Rehfuss et al., 1993; Gantz et al., 1993a,b; Mountjoy et al., 1994; Kishi et al., 2003; Bedenbaugh et al., 2022). Finally, with the exception of the MPOA and the ARC, there was very little co-expression of MC3R and MC4R mRNA in female Syrian hamsters in this study.

There have been multiple MC4R distribution studies – two in rats, one in mice, one in Atlantic salmon (Kalananthan et al., 2020), and one in the human hypothalamus (Siljee et al., 2013) – with a mixture of these studies having qualitative and quantitative data. There have been fewer studies mapping MC3R distribution. Each MC3R/MC4R distribution study has taken a different approach in creating a qualitative scale to compare levels of receptor expression. Aside from Bedenbaugh et al. (2022), the remaining studies did not explicitly state how they created their scale. For our study, a qualitative plus scale was derived from the average cell counts of neurons containing each receptor. The plus scale was used to compare the expression of MC3R and MC4R in hamsters with data reported for other species (Table 2). A plus scale was utilized for the MC3R distribution study conducted by Bedenbaugh et al. (2022). The plus scale was generated from the intensity color of MC3R expression, with each color having a natural break in the numbers of labeled cells (Table 2). One study did not generate an expression level scale (Gantz et al., 1993b) and we did not use that study in our comparison.

**Table 2:** Qualitative comparison of MC3R and MC4R hybridization across species.

Brain region	MC3R			MC4R		
	Hamster	Rat (Roselli-Rehfuss et al., 1993)	Mouse (Bedenbaugh et al., 2022)	Hamster	Rat (Mountjoy et al., 1994)	Rat (Kishi et al., 2003)
PrL	+		+ + + +	+ +		+
IL	−			+ +	+ +	+ +
NAc Core	−		+ +	+	+ +	+ +
NAc Shell	+			+		+ +
CPu medial	−		−	+	+ +	+ + + +
CPu lateral	−			+		
CPu dorsal	−			+		
OT	−			+ +	+ + +	+ + + +
LS	+ +		+ + +	+	+ + to + + + +	+ to + + + +
MS	+			+ +	+ +	+ +
BNST	+	+	+ +	+ +	+ + (+)	+
DEn	+			+ + +		
MePD	+		+ + + +	+ +	+ +	+ +
Dorsal CA1	+	+		+ +	+ (+)	+
Dorsal CA2	+	+		+ +	+ +	+
MD	+ + +		-	−	+	
LHb	+ +			+		+
MPOA	+ +	+ (+)	+	+ + + +	+ +	+
PVN	+	+ (+)	+ +	+ + +	+ to + + +	+ + to + + + +
VMH	+ + + +		+ + + +	+	+ to + + +	+
ARC	+ +	+ +	+ + +	+	+	+
Ventral CA1	+	+		+ + + +		+
PAG	+	+	+ +	+ + +	+ +	+ to + +
BIC	+ + + +			−		+ +
VTA	+	+ + (+)		+ + +	+	
DRN	+	+ + (+)	−	+ + +	+	+

Hamster MC3R and MC4R mRNA qualitative scales were determined by grouping the average number of cells per region into four plus sign descriptors. The scale is as follows: 0 average cell counts are categorized as lack of expression and equal to –, 1–24 average cell counts are categorized as low and equal to +, 25–49 average cell counts are categorized as moderate and equal to + +, 50–74 average cell counts are categorized as high and equal to + + +, and 75–100 average cell counts are categorized as highest and equal to + + + +.

The melanocortin system in the central nervous system is thought to be involved in a limited number of functions (Hill and Faulkner, 2017). Energy homeostasis is vital for the regulation of body weight, body temperature, and hormone balance among other processes. Melanocortin 3 receptors and MC4Rs regulate energy homeostasis and feeding behaviors (Ellacott and Cone, 2006). We observed staining for both receptors within hypothalamic and amygdalar regions that participate in these processes. The PVN, the VMH, and the ARC form a well-known pathway that regulates food intake and energy expenditure (Thomas et al., 2018). Energy homeostasis is regulated by proopiomelanocortin (POMC), neuropeptide Y (NPY), and Agouti-Related Protein (AgRP) expressing neurons in the ARC. POMC serves as a pro-peptide precursor for the melanocortin hormone. Further, AgRP is an endogenous receptor antagonist at MCRs that is expressed in NPY neurons in the ARC. The PVN receives input from POMC and AgRP expressing neurons in the ARC. All three of these regions were found to express MC3Rs and MC4Rs. Melanocortin 4 receptors in the PVN regulate body weight and food intake (Balthasar et al., 2005). Balthasar et al. (2005) hypothesized that specifically the parvocellular neurons of the PVN may be the mediators of food intake due to their projections to the nucleus of the solitary tract, which receives signals from the gut. The amygdala also has a role in regulating body weight (Balthasar et al., 2005). Melanocortin 3 receptor dependent roles in dopamine homeostasis and nutritional intake behaviors that occur within the VTA have been observed (Lippert et al., 2014; Dunigan et al., 2021b). Together, the expression pattern of MC3R and MC4R mRNA may be linked to the regulation of body weight.

There are species differences in MC3R mRNA expression in the PVN and the MePD of the amygdala, where the expression levels in hamsters are lower than that reported for the rat (Roselli-Rehfuss et al., 1993). The VMH of the hamster had similar expression levels of MC3R mRNA with that of the rat and mouse (Roselli-Rehfuss et al., 1993; Bedenbaugh et al., 2022). Melanocortin 3 receptor mRNA expression in the hamster ARC was equal to that in the rat and greater than the expression in the mouse (Roselli-Rehfuss et al., 1993; Bedenbaugh et al., 2022). The expression of MC4R within the PVN, the VMH, and the ARC in the hamster was similar to that in the rat, with rats having a range of expression depending on the subnuclei (Mountjoy et al., 1994; Kishi et al., 2003). Melanocortin 4 receptor expression levels within the MePD were equal in the hamster compared to the rat (Mountjoy et al., 1994; Kishi et al., 2003).

A secondary role of MC3Rs and MC4Rs includes sex behavior. Female hamsters serve as good animal models in investigating rewards derived from sex due to their immobility during lordosis (the primary rodent receptive posture), allowing researchers to isolate sex itself from other behaviors such as locomotion (Meisel et al., 1993). Erectile function in male rodents, leptin-stimulated hormone surges in female rodents, and grooming behaviors are all also mediated by the activity of MC3Rs and MC4Rs (Van der Ploeg et al., 2002; Pfaus et al., 2004; Tao, 2010). Lordosis can be inhibited or facilitated by alpha-MSH, an MC3/4R agonist; alpha-MSH receives signaling from estradiol to activate the neural pathways required for lordosis (Pfaus et al., 2004; Tsukahara et al., 2014). Pathways between the ARC, VMH, and LS are important for inhibiting lordosis behavior (Tsukahara et al., 2014). AgRP projections to the MPOA can stimulate the release of gonadotropin releasing hormone from the hypothalamus. The MPOA, BNST, and the PAG are involved in precopulatory behaviors such as odor preference and scent marking (Hennessey et al., 1992; Martinez and Petrulis, 2013). The expression of MC3R mRNA was greater than the expression of MC4R mRNA in the VMH and the LS, whereas the expression of MC3R and MC4R mRNA was equal in the ARC. Melanocortin 4 receptor mRNA expression was greater than the expression of MC3R mRNA in the MPOA, the BNST, and the PAG. This distribution of MC3R and MC4R expressing neurons may give insight into which receptor is more functionally represented in certain pathways.

The BNST and the PAG of the hamster had equal expression levels of MC3R mRNA with that of the rat whereas both regions along with the LS of the hamster had lower levels of MC3R mRNA expression compared to that of the mouse (Roselli-Rehfuss et al., 1993; Bedenbaugh et al., 2022). The MPOA of the hamster had higher levels of MC3R expression than that in the rat and mouse. The MC4R expression in the MPOA and PAG was higher in the hamster than that in the rat (Mountjoy et al., 1994; Kishi et al., 2003). The LS and the BNST of the hamster fall within the lower range of MC4R mRNA expression levels compared to the rat (Mountjoy et al., 1994; Kishi et al., 2003).

Studies have identified the involvement of MC3Rs and MC4Rs in reward circuits. Antagonists acting on MC3Rs and MC4Rs result in alterations in reward behaviors and locomotor activity (de Vrind et al., 2021; Dunigan et al., 2021a). A reward is derived from sex behaviors and regions such as the NAc, CPu, and the VTA are involved in the reward circuit. Injections into the VTA of agonists that are more selective for MC3R result in an increase in the release of dopamine in the NAc (Torre and Celis, 1988). In the NAc, CPu, and VTA, the expression of MC4R mRNA was greater than the expression of MC3R mRNA. The VTA has been previously observed as a region of high MC3R expression, though we observed low hybridization of MC3R in our hamsters. This may be due to differences in feeding behavior where hamsters maintain a rigid daily feeding schedule with feeding in compared to mice and rats where feeding is more of a motivated process (Schneider et al., 2002).

The MC3R mRNA expression levels of the hamster VTA are lower than that reported for the rat (Roselli-Rehfuss et al., 1993). The hamster NAc core and shell had lower levels of MC3R mRNA expression, compared to that of the mouse (Bedenbaugh et al., 2022). The CPu had a lack of MC3R expression across both the hamster and mouse. Nucleus accumbens core, shell, and CPuMC4R mRNA expression levels were lower in the hamster when compared to levels in the rat (Mountjoy et al., 1994; Kishi et al., 2003). The VTA of the hamster had higher MC4R expression than that of rats (Mountjoy et al., 1994; Kishi et al., 2003). Overall, the different brain regions have segregated subdivisions with different circuitry. Further, each function (metabolism, feeding, female sexual behavior, reward) has varied components, which may be mapped differently on the varied neural circuits.

Primary functions of melanocortin receptors are feeding and sex behavior, which was part of the basis for our focus on the forebrain and midbrain distribution of melanocortin receptors. Feeding circuits also involve hindbrain regions, suggesting that melanocortin receptors could be located more caudally. Further, melanocortins affect pain regulation which is modulated by hindbrain and spinal cord circuits. In fact, prior studies have demonstrated MCR distribution in the hindbrain and spinal cord. Kishi et al. (2003) and Bedenbaugh et al. (2022) mapped MC4R and MC3R mRNA across nuclei of the pons, medulla, and spinal cord. Melanocortin 4 receptor mRNA expression in the hindbrain ranged from background density in the nucleus ambiguus to highest density in the dorsal motor nucleus of the vagus and parabrachial nucleus, areas regulating feeding (Kishi et al., 2003). There was low to high density of MC4R mRNA expression within the spinal cord. Melanocortin 3 receptor mRNA expression was absent in the dorsal motor nucleus of the vagus nerve whereas labeling density was greater in the spinal nucleus of the trigeminal nerve (Bedenbaugh et al., 2022). As melanocortin 4 receptors have been linked functionally to pain processing, MC4R antagonists could be used therapeutically to reduce trigeminal neuropathic pain (Korczeniewska et al., 2023).

This study was conducted in four ovariectomized female subjects. Consequently, we do not know if there are differences in the number of MC3R or MC4R expressing neurons across the female’s reproductive cycle or whether there are sex differences for Syrian hamsters. Previous studies have found quantitative sex differences in MC3R and MC4R expression as well as sex differences in function. In the VTA, activation of MC3R neurons results in a significant decrease in food intake for females but not males, whereas the inhibition of MC3R neurons significantly decreases food intake in males but not females (Dunigan et al., 2021b). A knockout of MC3R results in reduced locomotor activity in female mice (Chen et al., 2000). It has been found that female mice have a significantly greater number of MC3R cells in the principal nucleus of the BNST, anteroventral periventricular nucleus, and ventral premammillary nucleus while male mice have a significantly greater number in the ARC (Sweeney et al., 2021; Bedenbaugh et al., 2022). At the same time that genetic sex or reproductive variables such as estrous cycle can affect expression of MC3R or MC4R receptors, these are quantitative differences that do not impact the overall distribution of melanocortin receptive neurons in the forebrain or midbrain. As such, the distribution of neurons we describe in our female hamsters should generalize to male hamsters as well.

One limitation of our study is that many regions are large with heterogenous subdivision and circuits. The MCR cell counts across regions will certainly vary depending on the particular subdivision sampled and where the sampling lies in a rostral-caudal dimension. We intentionally matched our sampling within restricted regions across subjects to ensure that each region was analyzed at the same level. The utility of our study and prior mapping studies is that it points the way for investigators interested in a specific brain region to perform detailed analyses of receptor distribution and circuits. Liu et al. (2003) provide an example of this approach where they engineered transgenic mice that expressed GFP in neurons under the control of an MC4R promoter. Following an analysis of the distribution of MC4R expressing neurons, they focused on the paraventricular nucleus of the hypothalamus and dorsal motor nucleus of the vagus. In the paraventricular nucleus MC4R was expressed in thyrotropin-releasing hormone producing neurons, whereas in the dorsal motor nucleus of the vagus MC4R was expressed in cholinergic neurons. Further, the paraventricular neurons were demonstrated to be responsive electrophysiologically to a synthetic melanocortin agonist.

To conclude, this study has added novel insights into the distribution of MC3R and MC4R across the central nervous system of a non-murine rodent and demonstrates the high level of conservation of melanocortin receptors among rodent species. Further, our study has advanced our understanding of the co-expression of MC3R and MC4R cells in the rodent nervous system, paving the way for future functional analyses of the melanocortin system.

## Data availability statement

The original contributions presented in the study are included in the article/Supplementary material, further inquiries can be directed to the corresponding author.

## Ethics statement

The animal study was reviewed and approved by University of Minnesota Institutional Animal Care and Use Committee.

## Author contributions

MH, RM, and JB designed the research. MH, AK-J, AP, GF, NS, and JB performed research. MH wrote the paper. All authors contributed to the article and approved the submitted version.

## Funding

This work was supported by grants from the National Institutes of Health to RM (R01 HD100007 and R01 HD100007-03S1). JB was supported by an NIH Training Grant (T32 DA007234) awarded to Paul Mermelstein.

## Conflict of interest

The authors declare that the research was conducted in the absence of any commercial or financial relationships that could be construed as a potential conflict of interest.

## Publisher’s note

All claims expressed in this article are solely those of the authors and do not necessarily represent those of their affiliated organizations, or those of the publisher, the editors and the reviewers. Any product that may be evaluated in this article, or claim that may be made by its manufacturer, is not guaranteed or endorsed by the publisher.
