# Supplementary material for: Melanocortin receptor 3 and 4 mRNA expression in the adult female Syrian hamster brain
Source: Front Mol Neurosci. 2023 Feb 23;16:1038341. doi: 10.3389/fnmol.2023.1038341 (PMC9995703; doi:10.3389/fnmol.2023.1038341)
Supplement: Supplementary file 1 [file Presentation_1.zip › Supplemental Figure 2.docx]

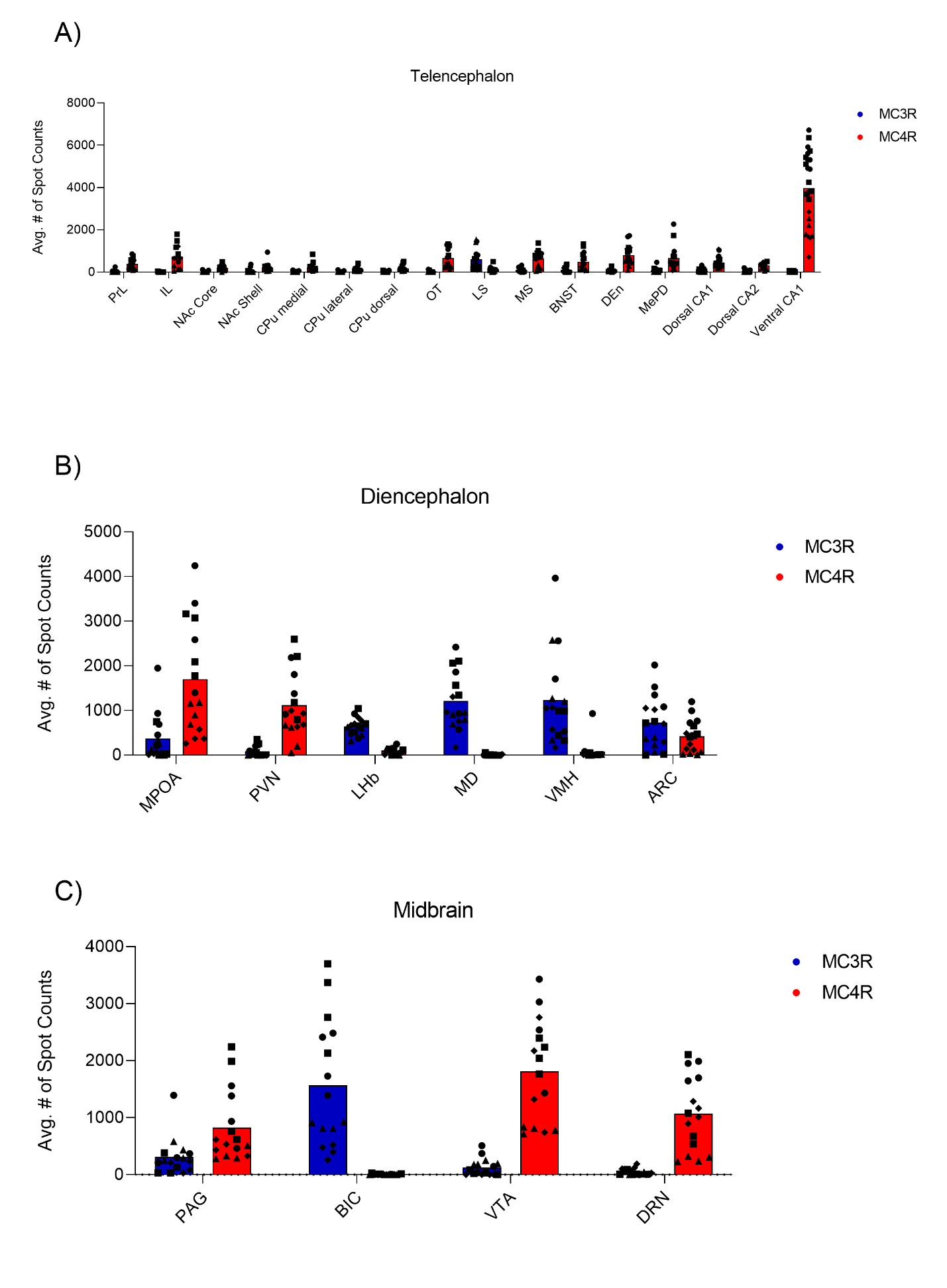


Supplemental Figure 2. The number of MC3R mRNA spots (blue left-hand bar for each pair) and MC4R mRNA spots (red right-hand bar for each pair) in the regions of the **(A)** telencephalon, **(B)** diencephalon, and **(C)** midbrain. Each symbol indicates a section of the region from each subject. Circles indicate counts from subject one. Squares indicate counts from subject two. Triangles indicate counts from subject three. Diamonds indicate counts from subject four.
