# Supplementary material for: Melanocortin receptor 3 and 4 mRNA expression in the adult female Syrian hamster brain
Source: Front Mol Neurosci. 2023 Feb 23;16:1038341. doi: 10.3389/fnmol.2023.1038341 (PMC9995703; doi:10.3389/fnmol.2023.1038341)
Supplement: Supplementary file 1 [file Presentation_1.zip › Supplemental Figure 3.docx]

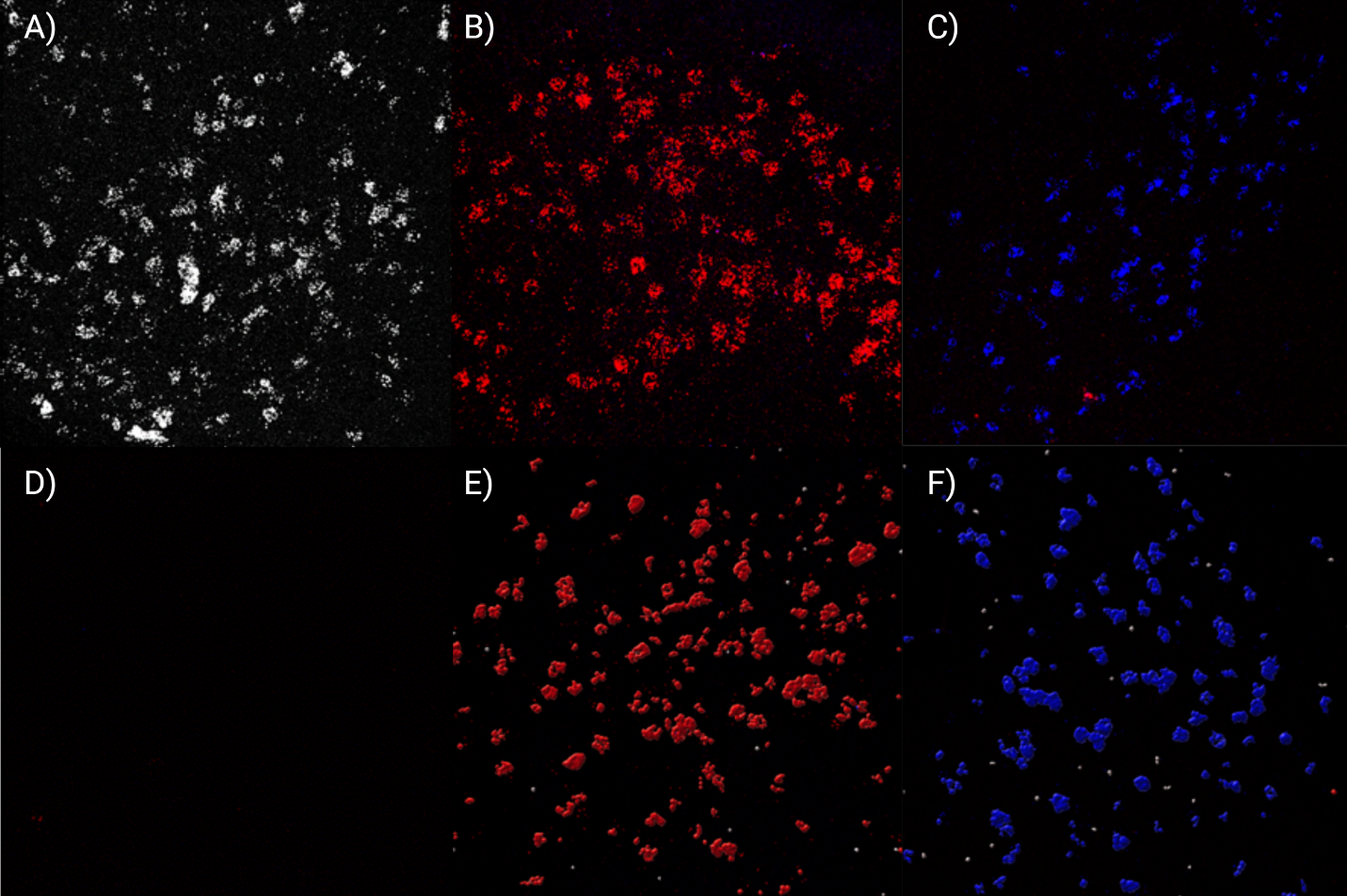


Supplemental Figure 3. **(A)** High magnification image of MC3R and MC4R mRNA expression without pseudo coloring. **(B)** High magnification image of MC4R mRNA expression with red pseudo coloring. **(C)** High magnification image of MC3R mRNA expression with blue pseudo coloring. **(D)** High magnification image demonstrating the absence of background expression within an area of the cortex that had no melanocortin receptor expression. **(E & F)** High magnification images of MC4R and MC3R mRNA expression with the surface counts overlay.
