# Supplementary material for: Melanocortin receptor 3 and 4 mRNA expression in the adult female Syrian hamster brain
Source: Front Mol Neurosci. 2023 Feb 23;16:1038341. doi: 10.3389/fnmol.2023.1038341 (PMC9995703; doi:10.3389/fnmol.2023.1038341)
Supplement: Supplementary file 1 [file Presentation_1.zip › Supplemental Figure 7.docx]

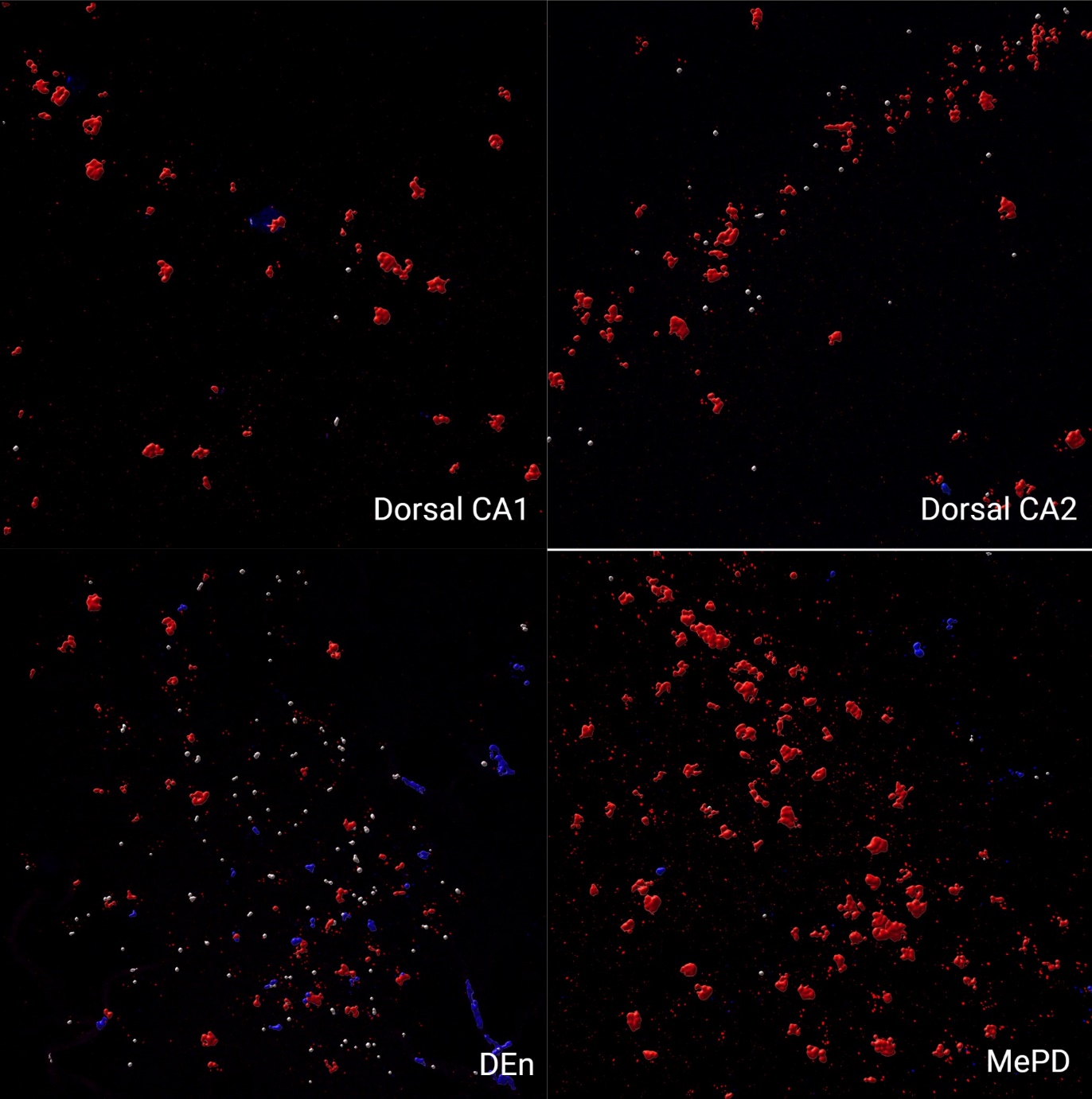


Supplemental Figure 7. The expression of MC3R and MC4R mRNA in the Dorsal CA1, Dorsal CA2, DEn, and MePD. Red labeling depicts MC4R mRNA positive surface counts. Blue labeling depicts MC3R mRNA positive surface counts. White labeling depicts expression that did not meet the criteria for surface counts. Created with BioRender.com
